# Elevated Procalcitonin as a Risk Factor for Postoperative Delirium in the Elderly after Cardiac Surgery—A Prospective Observational Study

**DOI:** 10.3390/jcm9123837

**Published:** 2020-11-26

**Authors:** Anna Kupiec, Barbara Adamik, Natalia Kozera, Waldemar Gozdzik

**Affiliations:** Department of Anesthesiology and Intensive Therapy, Wroclaw Medical University, Borowska 213, 50-556 Wroclaw, Poland; barbara.adamik@umed.wroc.pl (B.A.); natalia.kozera@student.umed.wroc.pl (N.K.); waldemar.gozdzik@umed.wroc.pl (W.G.)

**Keywords:** delirium, procalcitonin, elderly, functional decline, depression, cardiac surgery

## Abstract

One of the most common complications after cardiac surgery with cardiopulmonary bypass (CBP) is delirium. The purpose of this study was to prospectively investigate the risk of developing postoperative delirium in a group of elderly patients using a multivariate assessment of preoperative and intraoperative risk factors. A total of 149 elderly patients were included. Thirty patients (20%) developed post-operative delirium. Preoperative procalcitonin (PCT) above the reference range (>0.05 ng/mL) was recorded more often in patients who postoperatively developed delirium than in the non-delirium group (50% vs. 27%, *p* = 0.019). After surgery, PCT was significantly higher in the delirium than the non-delirium group: ICU admission after surgery: 0.08 ng/mL vs. 0.05 ng/mL *p* = 0.011), and for consecutive days (day 1: 0.59 ng/mL vs. 0.25 ng/mL, *p* = 0.003; day 2: 1.21 ng/mL vs. 0.36 ng/mL, *p* = 0.006; day 3: 0.76 ng/mL vs. 0.34 ng/mL, *p* = 0.001). Patients with delirium were older (74 vs. 69 years, *p* = 0.038), more often had impaired daily functioning (47% vs. 28%, *p* = 0.041), depressive symptoms (40% vs. 17%, *p* = 0.005), and anemia (43% vs. 19%, *p* = 0.006). In a multivariable logistic regression model, preoperative procalcitonin (odds ratio (OR) = 3.05), depressive symptoms (OR = 5.02), age (OR = 1.14), impaired daily functioning (OR = 0.76) along with CPB time (OR = 1.04) were significant predictors of postoperative delirium.

## 1. Introduction

In recent decades, we have seen a significant aging of the population in developed countries. Over 70% of people above the age of 60 suffer from cardiovascular disease [1]. Consequently, the number of elderly and very elderly patients undergoing cardiac surgery is increasing [2]. As the elderly are more burdened with comorbidities, this group is at high risk of perioperative complications, including death [3]. Therefore, perioperative care for elderly patients subjected to cardiac surgery becomes a big challenge for everyone—the surgeon, anesthesiologist, and intensivist—as is taking care of the patient after surgery. One of the most common complications after on-pump cardiac surgery, with reported incidence rates ranging up to 50%, is postoperative delirium, defined as fluctuating attention and awareness disturbances. Perioperative factors and the type of cardiac surgery are associated with postoperative delirium. In our previously published study, it was shown that cardiopulmonary hyperoxia episodes might be a risk factor associated with the occurrence of postoperative delirium [4]. Three subtypes of delirium have been differentiated: hypoactive, hyperactive, and mixed [5]. All of them have been associated with prolonged hospitalization, increased death rate [6], increased risk of intensive care unit (ICU) and hospital readmission [7], and possible long-term consequences such as lower quality of life or cognitive decline after the operation [8].

It is difficult to determine the origin of this complication as there are many potential systemic homeostasis disorders which can lead to delirium. Several hypotheses have been described, including neuroinflammation, oxidative stress, neuroendocrine dysregulation, circadian dysregulation, and neuronal aging [9]. Our knowledge of perioperative delirium risk factors in heart surgery is continuously growing, but there is still much that is unknown. The activation of an inflammatory response after surgery has been suggested as one of the possible mechanisms of delirium [9]. The use of cardiopulmonary bypass leads to the activation of a systemic inflammatory response and is associated with increased production of various inflammatory mediators during and after surgery [10,11]. An inflammatory marker, procalcitonin (PCT), may be considered a good predictor of postoperative complications early after cardiac surgery with cardiopulmonary bypass. High levels of PCT have been associated with postoperative complications such as infection [12], organ dysfunction [13], and increased mortality [14,15]. The usefulness of procalcitonin as a predictor of postoperative delirium caused by surgery and cardiopulmonary bypass (CBP) has not yet been investigated. Therefore, we have prospectively studied the risk of developing postoperative delirium in a group of elderly patients undergoing cardiac surgery with CPB, using a multivariate assessment of perioperative risk factors.

## 2. Experimental Section

The study was approved by the local Bioethics Committee (permission number 219/2016), and informed consent was collected from all patients participating in the study. Inclusion criteria were: planned cardiac surgery with CBP, age 65 years old or more, and full participation of the patient in the preoperative evaluation. Exclusion criteria were: inability to get informed consent, urgent operation, off-pump surgery, diagnosis of dementia before surgery, a different protocol of anesthesia (total venous anesthesia), or deep hypothermia procedures. 

### 2.1. Procalcitonin Measurement

In all patients, the PCT concentrations were routinely measured one day before surgery (baseline) and on day 0 (ICU admission after surgery), 1, 2, and 3 after surgery. All measurements were performed in the hospital laboratory (normal serum values are below 0.05 ng/mL) by using a chemiluminescent microparticle immunoassay (ARCHITECT B·R·A·H·M·S PCT, Abbott, Chicago, IL, USA). The limit of quantification of the test was 0.01 ng/mL, and the analytical sensitivity was 0.00 ng/mL.

### 2.2. Preoperative Assessment

A preoperative assessment was performed the day before the operation by a trained anesthesiologist and included the following evaluation of the patient’s ability to care for him- or herself with (I) an Activities of Daily Living scale (ADL); (II) an Instrumental Activities of Daily Living scale (IADL); an evaluation of the occurrence of cognitive decline using the (III) Mini-Mental State Examination score (MMSE); and an assessment of the occurrence of depression with an (IV) Geriatric Depression Scale (GDS-15). 

(I) ADL and (II) IADL are both parts of a comprehensive geriatric assessment [16] and are used to assess functional decline. The ADL questionnaire consists of 6 questions assessing basic functional activities as: (1) using the toilet, (2) controlling urination and defecation, (3) bathing, (4) eating, (5) getting out of bed, and (6) dressing oneself [17]. The ADL scale ranges from 0 to 6 pts., with 0 being the worst and 6 pts. being the best. A score of 6 indicates the full function and a score below 6 pts. indicates compromised daily functioning. The IADL consists of 8 questions about everyday activities: (1) to use a phone, (2) to do shopping, (3) to do housekeeping, (4) to handle finances, (5) to use a mode of transportation, (6) to be responsible for one’s own medication, (7) to prepare meals, and (8) to do laundry or craftwork [18]. The IADL score ranges from 8 to 24 points (1–3 pts. for each question), with 8 pts. being the worst and 24 pts. being the best. A score below 24 indicates compromised activities related to independent living.

As older populations are at risk of cognitive impairment, cognitive decline was measured with (III) the Mini-Mental State Examination (MMSE), one of the most common scores used for screening. It can also be easily used by physicians who are not psychiatrists. The MMSE assesses seven different cognitive domains: (1) orientation to time (range 0–5), (2) orientation to place (range 0–5), (3) word registration (range 0–3), (4) delayed recall (range 0–3), (5) working memory (range 0–5), (6) language (range 0–8), and (7) visuospatial (range 0–1). The total MMSE score ranges from 0 to 30 points. A score of 27–30 indicates no cognitive impairment and MMSE ≤ 26 indicates cognitive impairment [19].

In addition, we assessed depressive symptoms with (IV) a 15-point Geriatric Depression Scale GDS-15 with 0 pts. being the best and 15 pts. being the worst. A score ≥ 5 indicates depression with a sensitivity of 80% and specificity of 75% [20]. The GDS-15 score results were divided into two categories: no depression with GDS-15 < 5 and depressive symptoms present with GDS-15 ≥ 5. In addition, information on comorbidities was recorded. The European System for Cardiac Operative Risk Evaluation (Euroscore II) was used to predict the risk of death after heart surgery, as calculated by the available online Euroscore II calculator (euroscore.org). According to World Health Organization (WHO) criteria, preoperative anemia was defined, setting the hemoglobin cut off below 12 g/dL for females and 13 g/dL for males [21]. 

### 2.3. Anesthesia and Surgery

The induction of anesthesia was conducted with sufentanil, propofol, and rocuronium. Sufentanil and rocuronium infusion with inhaled sevoflurane was applied to maintain anesthesia. After administering heparin and achieving an activated clotting time longer than 480 s, CBP was initiated. A hollow fiber membrane oxygenator (Medtronic Affinity, Medtronic, Inc., Minneapolis, MN, USA) with an integrated polyester arterial line filter of 40 mm pore size and a roller pump with a non-pulsatile flow 2.4–2.5 L/min/m^2^ (Stockert S3, Sorin Group, Munich, Germany) was used in all patients. All patients were normothermic (37 °C). After the operation, patients were transferred to the ICU. Sedation with propofol was continued, and patients were extubated if they were hemodynamically stable, with sufficient oxygenation and without excessive bleeding. Postoperative pain was treated with Paracetamol 1 g every 6 h and Oxycodone 3–4 mg every 4 h. Procedure parameters and postoperative complications were noted, including the type of operation, aorta cross-clamping, CBP and operation time, and ICU stay and hospitalization lengths. Acute kidney injury was diagnosed according to Kidney Disease: Improving Global Outcomes (KDIGO) criteria [22].

### 2.4. Delirium Assessment

Patients were evaluated for delirium every 12 h, starting the morning after the operation for three subsequent days. We chose the Confusion Assessment Method for Intensive Care Unit (CAM ICU) to assess delirium. Its reported sensitivity is 83%, and specificity reaches up to 100% [23]. The CAM ICU is easy to perform and can be used by non-psychiatrists, physicians, and nurses. In our study, the CAM ICU assessment was performed by anesthesiologists trained for that purpose. The scale includes the evaluation of four features of delirium: fluctuating changes in mental status, inattention, an altered level of consciousness, and disorganized thinking.

The examination of consciousness was conducted with the Richmond Agitation-Sedation Scale (RASS). RASS is a 10-level score between −5 and +4 points, where −5 pts. indicates unarousable, 0 pts. indicates alert and calm, and +4 is combative. The CAM ICU can be used when the RASS ≥ −3. We used the RASS scale to identify subtypes of delirium. Hypoactive delirium was diagnosed in cases with −3 ≤ RASS ≤ 0 pts., and hyperactive delirium was diagnosed in patients with RASS > 0 pts. A mixed type of POD was diagnosed in the case of a hyper- and hypoactive type switching from one to another during the day.

### 2.5. Statistical Analysis

All analyses were performed with Statistica 13 software (StatSoft, Inc., Tulsa, OK, USA). Continuous variables were expressed as median (interquartile range between the 25th and 75th percentiles); categorical data were expressed as numbers and percentages. The distribution was not normal based on the Shapiro–Wilk test. Therefore, statistical analysis was performed using nonparametric tests. The Mann–Whitney U test was used for comparison of the continuous variables between study groups. A Friedman’s ANOVA with post-hoc tests was used to analyze changes within groups in the course of procalcitonin over time. Categorical variables were analyzed using the chi-square test; contingency tables were used to summarize the relationship between several categorical variables. Multivariate logistic regression analysis was performed to create a model predicting the development of postoperative delirium. The association between procalcitonin and selected covariates (age, gender, ADL, IADL, MMSE and GDS-15 scale, Euroscore II, anemia before surgery, CPB time, aorta cross clamp (AoX) time) and the development of postoperative delirium was assessed; the results were reported as odds ratios (OR) with 95% confidence intervals (CI). The first prepared model included preoperative and postoperative PCTs and all selected covariates. The collinearity of the variables was tested; two of the covariates (gender and MMSE) were collinear so those features were excluded from the analysis. The choice of the best model was proposed based on the backward selection of the model. From the baseline PCT, the PCT recorded on day 0, and the eight covariates (age, ADL, IADL, GDS-15 scale, Euroscore II, anemia before surgery, CPB time, AoX time), the procedure chose the best model with the baseline PCT and four covariates (age, IADL, depressive symptoms, and CPB time). All the tests were conducted with a 5% significance level. 

## 3. Results

### 3.1. Study Sample

**Figure 1:**
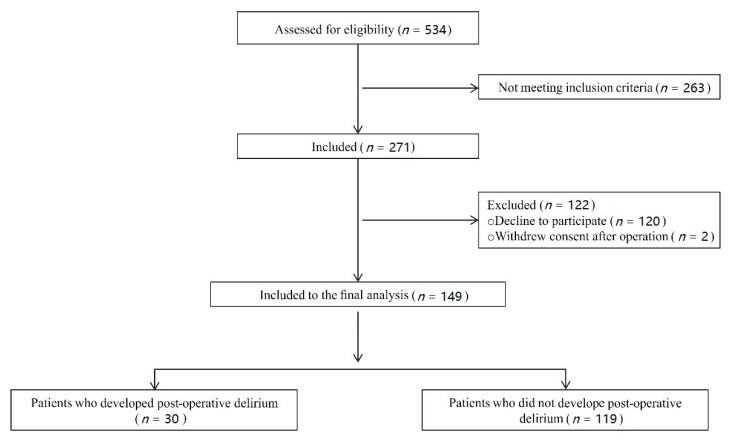
Flow diagram of the study.

A total of 534 consecutive patients who underwent cardiac surgery between January 2017 and December 2018 were screened for inclusion/exclusion criteria. Of this number, 271 patients met the inclusion criteria and were invited to the study. Finally, informed consent was obtained from 151 patients, but two patients withdrew their consent after the operation; 149 patients were included in the final analysis. The flow diagram of the study is presented in Figure 1.

None of the patients presented signs of infection before surgery, and the WBC count was within the reference range in both groups (with postoperative delirium: 7.37, interquartile range (IQR) 6.04–8.57 10^3^/mm^3^ vs. without delirium: 7.76, IQR 6.09–8.93 10^3^/mm^3^; *p* = 0.306).

**Figure 2:**
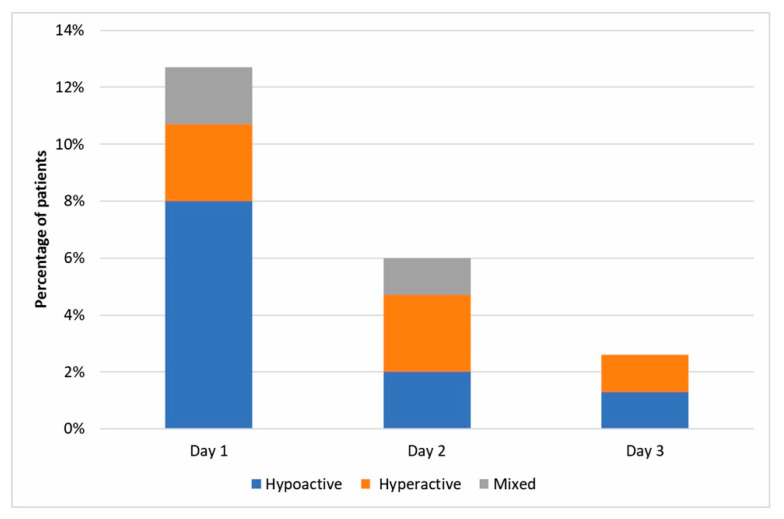
Postoperative delirium cases identified on days 1, 2, and 3 after surgery.

Thirty patients developed postoperative delirium (20%), and 119 patients (80%) did not. Hypoactive delirium was the most prevalent and was diagnosed in 50% (15/30) of patients. Hyperactive delirium was diagnosed in 33% (10/30) of patients, and 17% (5/30) of patients developed a mixed type of delirium (Figure 2). Of all delirium cases, 23 patients experienced delirium lasting less than 12 h, four patients between 12 and 24 h, and three patients experienced longer delirium. The majority of delirium (19 incidences) occurred on the first day postoperatively. Nine incidences were recorded on the second day, and five on the third day (Figure 2).

### 3.2. Risk Factors for Delirium

#### 3.2.1. Procalcitonin

**Figure 3:**
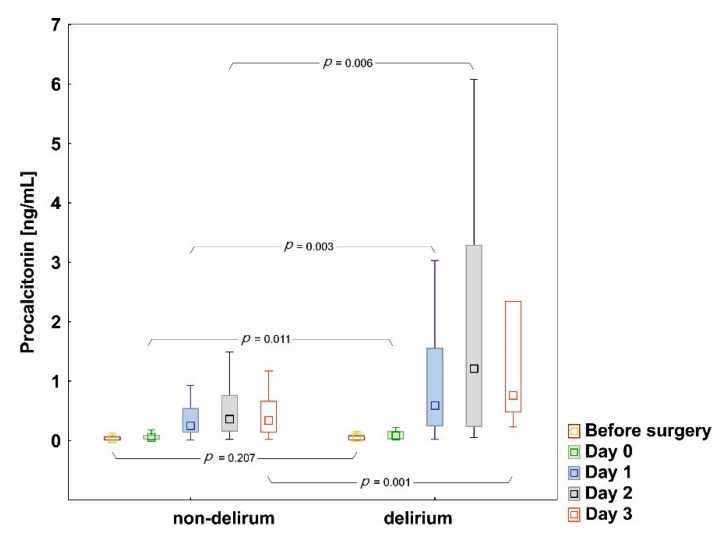
Procalcitonin concentrations in the group with and without postoperative delirium; *p*-values indicate statistically significant differences between groups; boxplot showing minimum and maximum (whiskers), median (middle point), and IQR values (box).

Preoperative (baseline) PCT levels above the reference range (>0.05 ng/mL) were found in 50% of patients who postoperatively developed delirium and in only 27% in the non-delirium group (*p* = 0.019). The comparison of PCT values between groups on each study day is presented in Figure 1. The median preoperative value of PCT was 0.05 ng/mL (IQR 0.01–0.08) in the delirium group and 0.03 ng/mL (IQR 0.01–0.06) in the non-delirium group (*p* = 0.207). After surgery, PCT levels were significantly higher in the delirium group than in patients without delirium on admission to the ICU (day 0: 0.08 ng/mL, IQR 0.03–0.15 vs. 0.05 ng/mL, IQR 0.02–0.09, *p* = 0.011) and for the consecutive days (day 1: 0.59 ng/mL, IQR 0.25–1.55 vs. 0.25 ng/mL, IQR 0.14–0.54, *p* = 0.003; day 2: 1.21 ng/mL, IQR 0.24–3.29 vs. 0.36 ng/mL, IQR 0.16–0.76, *p* = 0.006; day 3: 0.76 ng/mL, IQR 0.48–2.34 vs. 0.34 ng/mL, IQR 0.14–0.66, *p* = 0.001, respectively, in the delirium and non-delirium group), with a peak value on the second day after surgery (Figure 3). 

The analysis of changes in PCT concentrations overtime within the groups showed significant changes in the delirious group (*p* < 0.001, Friedman’s ANOVA test) and in the group without delirium (*p* = 0.021); post-hoc tests showed a significant increase of PCT on days 1 and 2 compared to the baseline in both groups. 

Three patients in the delirium group and two in the group without delirium had infection symptoms in the postoperative period; two patients were diagnosed with pneumonia, two with surgical-site infection, and one with pneumonia and a urinary tract infection. The other patients, despite an elevated PCT level after surgery, did not present any signs of infection.

#### 3.2.2. Age

The median age in the group of patients with postoperative delirium was higher than in the non-delirium group (74 years, IQR 70–76 vs. 69 years, IQR 67–74; *p* = 0.038) (Table 1). 

#### 3.2.3. Comorbidities

The distribution of comorbidities such as cerebrovascular disease, hypertension, atrial fibrillation, diabetes, or renal insufficiency was comparable in the study groups, and anemia was more than two times more common in the group of patients who experienced postoperative delirium compared to the group without delirium (43% vs. 19%, *p* = 0.006) (Table 1). 

#### 3.2.4. Euroscore II

As estimated by the pre-surgery Euroscore II risk model, the risk of death after cardiac surgery was higher in patients who eventually developed delirium postoperatively than in the non-delirious group (2.5% vs. 1.8%, *p* = 0.009). The observed mortality was 3.3% in the group with delirium and 0.8% in the non-delirium group (*p* = 0.323).

#### 3.2.5. Preoperative Geriatric Assessment Scores

MMSE: the overall scores were not different among patients with or without postoperative delirium (median 25 pts. in each group). An analysis of the MMSE domains between patient groups revealed only a statistically significant difference for the orientation to place domain (*p* = 0.020); results in other domains (orientation to time, word recall, working memory, language, and visuospatial) were similar in both study groups. A total of 39% of patients did not show signs of cognitive impairment (27%, 8/30 in the delirium group; 42%, 50/119 in the non-delirium group); MMSE ≤ 26, which indicates cognitive impairment, was found in 73% of patients (22/30) in the delirious group and in 58% (69/119) in the non-delirium group (*p* = 0.123). 

ADL: the majority of patients did not show signs of a decline in functional status (93%, 28/30 in the delirium group; 99%, 118/119 in the non-delirium group). Compromised daily functioning was detected in 2 patients in the delirious group (both cases with 2 pts.) and in 1 in the non-delirium group (5 pts.).

IADL: no sign of decline in functional status was recorded in only 53% of patients (16/30) in the delirium group and 72% (86/119) in the non-delirium group. Compromised daily functioning was detected in 47% of patients (14/30) in the delirium group and 28% (33/119) in the non-delirium group (*p* = 0.046). Among those with compromised daily functioning, patients with postoperative delirium were less independent in terms of shopping (*p* = 0.041), housekeeping (*p* < 0.001), using a mode of transportation (*p* = 0.014), being responsible for one’s own medication (*p* = 0.035), preparing meals (*p* = 0.022), and doing laundry (0.001) compared to the non-delirium group.

GDS-15 score: GDS-15 ≥ 5, indicating depressive symptoms, was detected in 12 patients in the delirious group (40%) and in 20 in the non-delirious group (17%) (*p* = 0.005). The median value of GDS-15 was 5 pts. (IQR 3–7) in the delirious group and 3 pts. (IQR 2–5) in patients without delirium (*p* = 0.003).

An analysis comparing the preoperative geriatric characteristics of subjects who developed delirium and those who did not is presented in Table 2.

#### 3.2.6. Surgery and Postoperative Indices

In the group with postoperative delirium, a complex procedure was used more often (40% vs. 14%, *p* = 0.009). As expected, cardiopulmonary bypass and aortic cross-clamp time were significantly longer in patients who eventually developed delirium than in the group without delirium, but anesthesia time was similar in both study groups (Table 3). The postoperative analysis showed that in the group of patients with delirium, respiratory support was significantly longer than in the group without delirium (290 vs. 200 min, *p* = 0.002), acute kidney injury was diagnosed three times more often (20% vs. 7%, *p* = 0.025), the hospital stay was longer (11 vs. 9 days, *p* = 0.002), and mortality was higher (3.3% vs. 0.8%, *p* = 0.323) (Table 3).

### 3.3. Prediction Model of Postoperative Delirium

Multivariate logistic regression analysis was performed to create a model to predict the development of postoperative delirium. The model’s backward selection determined the choice of the variables from the set of perioperative parameters (procalcitonin, age, gender, ADL, IADL, MMSE, and GDS-15 scale, Euroscore II, anaemia before surgery, CPB time, AoX time). The baseline procalcitonin (OR = 3.05; IQR 1.01–9.19), GDS-15 ≥ 5 indicating depressive symptoms (OR = 5.02, IQR 1.67–15.10), age (OR = 1.14; IQR 1.02–1.26), IADL score (OR = 0.76; 0.63–0.91), and CPB time (OR = 1.04; IQR 1.02–1.06) were significant predictors of postoperative delirium; other parameters did not enter the model. The results are presented in Table 4.

## 4. Discussion

The key finding of this study is that elevated preoperative procalcitonin, along with other preoperative factors such as age, depressive symptoms, and compromised daily functioning, accompanied by prolonged CBP time, are the most critical factors in the development of postoperative delirium after cardiac surgery. A multivariate logistic regression model was used to estimate the strength of the association of these parameters with the development of postoperative delirium. To our knowledge, this is the first study showing the usefulness of procalcitonin as a predictor of the development of postoperative delirium in patients undergoing cardiac surgery. 

PCT is a peptide precursor of calcitonin and a biomarker of inflammation. In ICU patients, PCT has been used to identify bacterial infections and monitor antibiotic therapy as part of standard care for septic patients. However, there is growing evidence that pre-procedural PCT may be a marker that also predicts an adverse outcome in non-infectious diseases. Keranov et al. recently investigated the relationship between preoperative PCT and postoperative outcome in patients after transcatheter aortic valve implantation (TAVI). Multivariable analysis revealed that preoperative PCT was an independent predictor of 30-day mortality (HR 2.84) and 1-year mortality (HR 1.90); significantly higher mortality was observed in the group in which the preoperative PCT value was only slightly above the reference range (≥0.06 ng/mL) [24]. The importance of preoperative procalcitonin as a predictor of delirium has not yet been established. In the present study, it was observed that 50% of patients who developed postoperative delirium and only 27% of patients without delirium had procalcitonin above the reference range prior to surgery and according to the multivariate logistic regression analysis, preoperative PCT significantly increased the risk of postoperative delirium with an odds ratio of 3.05 (*p* = 0.048).

The causes of delirium are multifactorial and in our study the best multivariable model for predicting delirium development after cardiac surgery included the preoperative PCT concentration along with other factors such as IADL score, depressive symptoms, age, and the duration of CPB. The elderly are particularly sensitive to the development of postoperative delirium; in addition, delirium duration is longer in elderly patients than in younger patients subjected to cardiac surgery. Previously, Cereghetti et al. found that each year of life significantly increased postoperative delirium risk with an OR of 1.06 [25]. These results coincide with our research; according to the multivariate logistic regression analysis, the patient’s age was a significant factor of the model that predicted postoperative delirium with an odds ratio of 1.13.

Depression is frequent in older people and seems to be another critical risk factor of postoperative delirium. We reported here that the GDS-15 ≥ 5, indicating depressive symptoms, was associated with a 5-fold increased risk of postoperative delirium. A study by Eshmawe et al. confirmed that a higher preoperative depression score was associated with an increased risk of postoperative delirium [26]. In another study, Oldham et al. demonstrated a relationship between the occurrence of preoperative depression and the development and severity of delirium after CABG. The authors used three different scales to diagnose depression, and only one scale (Patient Health Questionnaire 9) was predictive of delirium, while the results of two other scores (GDS-15 and HDRS) were shown to have little or no association with postoperative delirium [27]. Different scales have been used to measure depression and it remains unclear which measure of depression may be most predictive of outcome. 

Advanced age is one of the most important risk factors for functional deterioration assessed by the IADL score. It was estimated that 40% of men and more than 50% of women were limited in at least one IADL activity [28]. A decline in physical activity such as doing housework, travel, and shopping were more age-dependent than cognitive activities such as using a telephone, managing finances, or taking one’s medication [29]. In our research, patients who developed postoperative delirium had significantly compromised physical activities assessed with the IADL score. Moreover, in the multivariate logistic regression analysis, a lower IADL score estimated before surgery was a significant risk factor for postoperative delirium. These results confirm the importance of examining depression or the deterioration of physical and cognitive activity before an operation in an elderly patient. As part of a comprehensive geriatric assessment, tools such as the IADL and GDS-15 should be a routine part of managing an elderly population to help identify cases of increased risk of postoperative delirium. 

In this study, we found other differences in the analyzed parameters between the delirious and non-delirious group, but these variables were not significant in the multivariable logistic regression model. One of these variables was the postoperative PCT concentration. We diagnosed postoperative infection in only 5 patients, but the increased PCT was significant in both the delirium and non-delirious groups. This can be explained by the fact that the concentration of procalcitonin may rise in the course of the inflammatory reaction in response to surgical trauma without infection, and a transient increase in the PCT concentration has been observed even during uncomplicated heart surgery [30]. In cardiac surgery, the systemic inflammatory response can be activated by many factors such as surgical trauma, CBP circuit, endotoxemia, and ischemia-reperfusion injury. Various inflammatory mediators, including PCT, are released and lead to systemic effects such as vasodilatation and disturbances in the microcirculation [10]. Long CBP is a powerful inductor of a systemic inflammatory reaction, and brain function may also be affected. Consistent with the neuroinflammatory hypothesis of postoperative delirium, inflammatory mediators released when the systemic inflammatory response has been activated cross the blood-brain barrier, activating microglial cells to produce inflammatory mediators. Consequently, damage to brain tissue, dysfunction of neuron activity, disturbances in the neurotransmitter system, impaired synaptic conduction, and leaking of intercellular connections of blood–brain barrier cells were detected [9,31].

Here, we assessed PCT because daily PCT measurements are readily available as part of routine in-hospital monitoring, and the predictive value of PCT for diagnosing complications after cardiac surgery had been previously confirmed. Clementi et al. presented results that the PCT measurement performed 48 h after the cardiac operation was a good predictor of postoperative renal and respiratory complications [14]. In another study, Klingele et al. showed that a single PCT measurement taken the day after surgery was a predictor of delayed complications such as prolonged hospitalization, ICU readmission, and hospital death [32].

The importance of postoperative procalcitonin as a predictor of delirium has not yet been established. Earlier, McGrane et al. demonstrated that in a population of non-cardiac ICU patients, high PCT values recorded on ICU admission predicted prolonged periods of acute brain dysfunction, linking inflammation as an essential mechanism in the pathophysiology of delirium [33]. In a later prospective study by Nemeth et al., the relationship between changes in the procalcitonin concentration measured on the first day after surgery and the occurrence of postoperative cognitive dysfunction was assessed in a population of elderly patients undergoing on-pump cardiac surgery, and no relationship was found between inflammatory response and cognitive dysfunction [34]. In this study, the median level of PCT was elevated after the operation with a peak value on the second day, and it was significantly higher in patients with postoperative delirium than in those without delirium. However, it should be emphasized, that while median PCT level was elevated in patients throughout the postoperative period, in multivariate logistic regression analysis only pre-operative PCT along with age, depression, daily functioning dysfunction, and CBP were significant in the delirium prediction model. As PCT tests are readily available (hospital laboratory, point of care testing), fast, and relatively cheap, it may be an additional indicator useful for the early identification of patients at risk for postoperative delirium.

Comorbidities are known risk factors for the development of postoperative delirium [35]. In our study, a majority of patients were diagnosed with arterial hypertension; diabetes and chronic renal insufficiency were common. Except for anemia, there was no difference in the distribution of comorbidities between groups. Anemia was diagnosed in 43% of patients in the group who developed postoperative delirium, and only 19% in the group without delirium. Previous studies have shown that preoperative anemia was considered a risk factor for the outcome in non-cardiac surgery; it was associated with a more extended hospital stay, a higher risk of perioperative complications, and higher mortality [36]. In a recent study of 800 patients undergoing elective non-cardiac surgery, anemia was associated with postoperative delirium and longer hospitalization [37]. The impact of preoperative anemia on postoperative delirium in the cardiosurgical population is not yet well understood, and the results published so far have often been contradictory. In a systematic review based on 34 studies, several studies identified anemia as an important risk factor for delirium following on-pump cardiac surgery, while others did not find an association [38]. In a recently published study by Smulter et al., the preoperative hemoglobin level was not associated with postoperative delirium [39]. In our study, preoperative anemia was more than twice as common in the group with postoperative delirium than in patients without delirium (43% vs. 19%, *p* = 0.006); however, in a multivariate logistic regression analysis, the presence of preoperative anemia was not included in the prediction model. It seems that this issue requires further evaluation in larger populations. 

In the present study, more patients who postoperatively developed delirium required a complex procedure. This resulted in a significant prolongation of CBP time. Along with long aortic cross-clamping, prolonged CBP time has been one of the most important factors of postoperative delirium [6]. This observation was also confirmed in our study in the multivariate logistic regression analysis. The duration of CPB was a significant factor in the model predicting postoperative delirium with an odds ratio of 1.04. This observation leads to the conclusion that qualifying elderly patients for long and complex procedures require careful consideration and balancing the risks and potential benefits. 

Our study has several limitations. First, we did not assess the long-term consequences of delirium, postoperative cognitive decline, or a reduced quality of life after the operation. Second, our model was based on patients in one center and the study sample was relatively small. Multi-centered research on the assessment of procalcitonin and other inflammatory markers is worth considering.

## 5. Conclusions

The multivariate logistic regression model shows that preoperative factors such as procalcitonin above a normal range, older age, depressive symptoms, impaired functional status, along with the intraoperative factor of a longer cardiopulmonary bypass time are associated with an increased risk of developing postoperative delirium. Further research is needed to confirm our observations on a larger cohort of patients.

## Figures and Tables

**Table 1:** Preoperative characteristics: an analysis comparing characteristics of subjects who developed delirium and those who did not.

Preoperative Characteristics	Delirium	Non-Delirium	*p*
	*n* = 30	*n* = 119	
Age	73.5 (70–76)	69 (67–74)	0.038
Gender, men, *n* (%)	18 (60)	79 (66)	0.511
BMI, (kg/m^2^)	28.4 (24.2–29.4)	27.5 (25.3–31.0)	0.597
Euroscore II, (%)	2.5 (1.6–5.7)	1.8 (1.2–2.8)	0.009
Comorbidities, *n* (%)			
Cerebrovascular disease	7 (23)	13 (10)	0.138
Arterial hypertension	26 (86)	107 (89)	0.607
Diabetes mellitus	11 (36)	44 (36)	0.975
Continuous atrial fibrillation	2 (6)	11 (9)	0.654
Chronic renal insufficiency	8 (26)	20 (16)	0.216
Anemia, *n (%)*	13(43)	23 (19)	0.006
Ejection fraction, *n* (%)	60 (50–65)	60 (50–65)	0.471
Nicotine abuse, *n* (%)	6 (20)	26 (22)	0.825

Data are presented as median (interquartile range) or percentage. BMI: Body Mass Index.

**Table 2:** Preoperative geriatric assessment: an analysis comparing the assessment of subjects who developed delirium and those who did not.

	Delirium	Non-Delirium	*p*
	*n* = 30	*n* = 119	
**MMSE, *n* (%)**			0.123
no cognitive impairment (≥27 pts.)	8 (27)	50 (42)	
cognitive impairment (≤26 pts.)	22 (73)	69 (58)	
**ADL, *n* (%)**			0.103
full functioning (6 pts.), *n* (%)	28 (93)	118 (99)	
compromised functioning (<6 pts.), *n* (%)	2 (7)	1(1)	
**IADL, *n* (%)**			0.046
full functioning (24 pts)	16 (53)	86 (72)	
compromised functioning (total score < 24 pts.):			
(1) use phone	2 (7)	3 (3)	0.471
(2) shopping	5 (16)	6(5)	0.041
(3) housekeeping	9 (30)	4 (4)	<0.001
(4) handle finances	7 (23)	23 (19)	0.235
(5) a mode of transportation	10 (33)	15(12)	0.014
(6) responsibility for own medication	5 (16)	5 (5)	0.035
(7) preparation of food	5 (16)	4 (3)	0.022
(8) doing laundry	7 (23)	5 (5)	0.001
**GDS-15, *n* (%)**			0.005
no depression (<5 pts.)	18 (60)	99 (83)	
depressive symptoms (≥5 pts.)	12 (40)	20 (17)	

Data are presented as median (interquartile range) or percentage. MMSE: Mini Mental State Examination; ADL: Activities of Daily Living; IADL: Instrumental Activities of Daily Living; GDS-15: 15-Item Geriatric Depression Scale.

**Table 3:** Perioperative characteristics: an analysis comparing the characteristics of subjects who developed delirium and those who did not.

	Delirium	Non-Delirium	*p*
	*n* = 30	*n* = 119	
**Intra-operative characteristics**			
Type of operation, *n* (%)			0.009
CABG	16 (53)	84 (71)	
Valve only	2 (7)	18 (15)	
CABG + valve	12 (40)	17 (14)	
CBP time (min)	102 (81–127)	79 (67–94)	<0.001
AoX time (min)	57 (43–81)	43 (34–60)	0.004
Anesthesia time (min)	272 (240–305)	255 (235–280)	0.101
**Post-operative characteristics**			
Respiratory support (minutes)	290 (210–525)	200 (175–270)	0.002
AKI, *n* (%)	6 (20)	8 (7)	0.025
ICU stay (days)	3 (2–6)	3 (2–5)	0.133
Hospital stay (days)	11 (10–15)	9 (8–11)	0.002
In-hospital mortality, *n* (%)	1 (3.3)	1 (0.8)	0.323

Data are presented as median (interquartile range) or percentage. CBP: cardiopulmonary bypass; AoX: aorta cross clamp; AKI: acute kidney injury; ICU: intensive care unit.

**Table 4:** Univariate and multivariate logistic regression analysis of the predictors of delirium.

	Univariate Analysis			Multivariate Analysis		
	Odds Ratio	95% CI	*p*	Odds Ratio	95% CI	*p*
**PCT baseline**	2.70	1.15–6.32	0.022	3.05	1.01–9.19	0.048
**IADL**	0.81	0.70–0.92	0.002	0.76	0.63–0.91	0.003
**Depressive symptoms**	3.30	1.37–7.91	0.008	5.02	1.67–15.10	0.004
**Age**	1.08	1.01–1.17	0.031	1.14	1.02–1.26	0.016
**CPB time**	1.02	1.01–1.04	<0.001	1.04	1.02–1.06	<0.001
**AoX time**	1.02	1.01–1.04	0.003			
**Euroscore**	1.21	1.06–1.37	0.004			
**Gender**	0.75	0.33–1.73	0.511			
**MMSE**	0.96	0.84–1.09	0.595			
**ADL**	0.34	0.10–1.16	0.086			
**Anemia before surgery**	3.19	1.35–7.49	0.007			

CI: confidence interval; IADL: Instrumental Activities of Daily Living; CBP: cardiopulmonary bypass; PCT: procalcitonin; AoX: aortic cross clamp; MMSE: Mini Mental State Examination; ADL: Activities of Daily Living.
